# Regulation of E2F1 Transcription Factor by Ubiquitin Conjugation

**DOI:** 10.3390/ijms18102188

**Published:** 2017-10-19

**Authors:** Laurence Dubrez

**Affiliations:** 1 Université de Bourgogne Franche-Comté, LNC UMR1231, 21000 Dijon, France; ldubrez@u-bourgogne.fr; Tel.: +33-380-393-356; 2 Institut National de la Santé et de la Recherche Médicale (Inserm), LNC UMR1231, 21000 Dijon, France

**Keywords:** E2F1, ubiquitination, cell cycle, DNA damage

## Abstract

Ubiquitination is a post-translational modification that defines the cellular fate of intracellular proteins. It can modify their stability, their activity, their subcellular location, and even their interacting pattern. This modification is a reversible event whose implementation is easy and fast. It contributes to the rapid adaptation of the cells to physiological intracellular variations and to intracellular or environmental stresses. E2F1 (E2 promoter binding factor 1) transcription factor is a potent cell cycle regulator. It displays contradictory functions able to regulate both cell proliferation and cell death. Its expression and activity are tightly regulated over the course of the cell cycle progression and in response to genotoxic stress. I discuss here the most recent evidence demonstrating the role of ubiquitination in E2F1’s regulation.

## 1. Introduction

E2F1 (E2 promoter binding factor 1) is the founding member of an evolutionarily conserved family of transcription factors that play critical roles in the regulation of the cell cycle and apoptosis. It was identified in 1987 as a cellular factor able to bind a sequence element in the adenovirus E2 promoter [1,2,3]. Research into its cellular functions and regulation mechanisms soon led to the finding that E2F1 is a downstream target of the pocket protein Retinoblastoma (Rb) [4,5,6,7]. This discovery linked E2F1 to the cell cycle and paved the way for extensive studies aimed at determining the role of E2F1 in cell cycle regulation, analyzing regulatory mechanisms that control E2F activity and identifying E2F target genes. At present, the E2F family contains 10 members characterized by a highly conserved DNA binding domain (DBD).

The E2F family members are generally classified into transcriptional activators (E2F1, E2F2, E2F3a and E2F3b), repressors (E2F4 and E2F5), and inhibitors (E2F6, E2F7a, E2F7b and E2F8). However, this simplistic classification does not necessarily reflect the exact activity of E2Fs. Deletion and knock-in strategies in insects and mice reveal significant functional redundancies among activators and repressors that make the functional analysis of E2F family members difficult (for review, see [8]). Moreover, the complexity is increased by the presence of cross-regulation mechanisms in order to balance the expression of activator and repressor members in the course of the cell cycle progression. E2Fs contribute to the timely sequence of the cell cycle phases. E2F activators and repressors are controlled by pocket proteins, including Rb, p107, and p130. Schematically, in quiescent cells, gene promoters are occupied by the repressors E2F4 and E2F5, associated with the Rb-related pocket proteins p107 or p130, while E2F activators are inactivated by binding to Rb. Phosphorylation of the pocket proteins by cyclin/cyclin dependent kinases (CDKs upon mitotic stimulation releases E2Fs. The free E2F activators can then initiate the transcription of E2F target genes, driving G1 to S phase transition while E2F4 and E2F5 are shuttled to the cytoplasm. In G2, target gene expression is restricted by the recruitment on promoters of the inhibitors E2F6, E2F7 and E2F8, independently of the pocket proteins. E2F inhibitors have also been reported to restrain activator E2F function by directly repressing gene expression [8].

Among E2F family members, E2F1 is probably the most studied and its regulatory mechanisms are starting to be relatively well understood. E2F1 expression and activity fluctuate in a cell-cycle-dependent manner, both at a transcriptional and a translational level. E2F1 is also rapidly upregulated in response to DNA damage, mediated by viral infection [2] or by various endogenous or exogenous stimuli. Paradoxically, E2F1 was shown to be able to promote the transcription of genes involved in cell proliferation, as well as genes involved in cell death. Other identified E2F targets include genes related to DNA damage response, angiogenesis, and growth factors signaling pathways [9]. Moreover, non-transcriptional activities of E2F1 have been described, such as the ability to favor apoptosis [10]. The E2F1/Rb axis is dysregulated in a large proportion of human cancers, but accordingly to the dual activity of E2F1, this represents either a good or a bad prognostic hallmark. Because of its paradoxical activity and its involvement in cancer, deciphering the regulation mechanisms of E2F activity is an important challenge and has been the subject of intensive research.

Several levels of regulation have been described [11]. The best known is regulation by a direct interaction with RB family members that repress the transcriptional activity of E2F1. The E2F–Rb interaction is dependent on the phosphorylation status of Rb. Other important partners are its co-factor, dimerization partner 1 (DP1), which is critical to the high-affinity binding of the transcription factors to DNA, and cyclin A, which is required for the cyclic regulation of E2F1 along the cell cycle. Besides these three major regulators, a number of co-factors and co-regulators have been described; most of them are protein-modifying enzymes. Many post-translational modifications, including phosphorylation, acetylation, methylation, neddylation, sumoylation, and ubiquitinylation, regulate the stability, the protein interacting pattern, the activity, and the recruitment of E2F1 on specific target gene promoters [11]. This review will present an overview of the regulation of E2F1 by ubiquitin conjugation modifications.

## 2. Ubiquitination

Ubiquitination consists of a reversible covalent binding of ubiquitin moieties on a lysine residue of a target protein. Ubiquitin contains seven lysine residues that, along with the amino-terminal methionine, are acceptor residues for ubiquitin-forming poly-ubiquitin chains of different topologies. One protein can be modified by a single ubiquitin molecule or ubiquitin chains on one or more lysine residues. These ubiquitin moieties are specifically recognized by ubiquitin-binding domains (UBDs) containing effector proteins that activate downstream events such as proteasomal degradation, signaling platform assembly, conformational change, or recruitment to a specific subcellular location. For example, K48- and K11-linked ubiquitin chains are signals for proteasomal degradation, whereas K63-linked ubiquitin chains more often generate signals for activation, subcellular localization modification, or recruitment to dedicated signaling platforms. Ubiquitin moieties can be removed by deubiquitylases (DUBs). Thus, ubiquitination represents a very fast, dynamic mechanism for regulating the cell fate of intracellular proteins (for review, see [12,13]). The conjugation of ubiquitin or ubiquitin chains to a target protein occurs through a three-step multi-enzymatic reaction involving an E1-ubiquitin-activating enzyme, an E2-conjugating enzyme, and an E3-ubiquitin ligase. It is generally admitted that E2 defines the nature of ubiquitin chains, while E3 supports substrate recognition.

## 3. Cell Cycle Regulation of E2F1 by Ubiquitination

**Figure 1:**
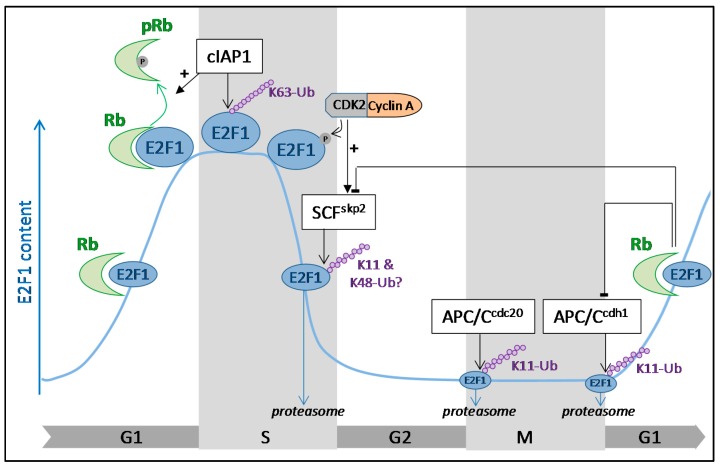
Cell cycle regulation of E2F1 (E2 promotor binding factor 1) by ubiquitination. The E2F1 cellular content fluctuates along the cell cycle. It progressively increases in G1, peaks in S phase to promote the expression of genes required for the S-phase transition, then declines as cells progress through the S phase of the cell cycle. In G1, Rb (retinoblastoma) binds to E2F1 and represses its transcriptional activity. Sequestration of E2F1 by Rb stabilizes the protein and allows its accumulation during the G1 phase. The stability of E2F1 is controlled by ubiquitination throughout the cell cycle. In S phase, E2F1 is conjugated with K63-linked ubiquitin chains in a cIAP1 (cellular inhibitor of apoptosis 1)-dependent manner, contributing to its stabilization. At the end of the S phase, phosphorylation of E2F1 by CDK2 (cyclin-dependent kinase 2)/cyclin A complex is a signal for SCF (S-phase kinase-associated protein 1-cullinnn1-F-box)^skp2^-mediated ubiquitination that targets E2F1 for proteasomal degradation. In the G2, M, and early G1 phases of the cell cycle, E2F1 content is maintained at low levels thanks to APC/C (anaphase promoting complex/cyclosome)^cdc20^ and APC/C^cdh1^-mediated ubiquitination and proteasomal degradation.

**Figure 2:**
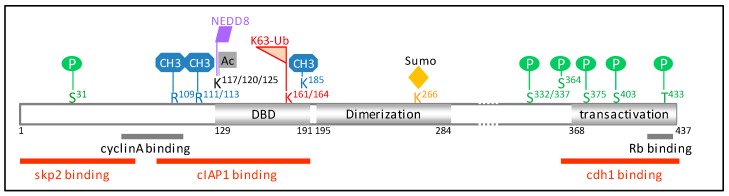
Schematic representation of E2F1 protein sequence (NP_005216.1). The regions interacting with cyclin A, Rb (retinoblastoma), and the E3-ubiquitine ligases skp2 (S-phase kinase-associated protein 2), cIAP1 (cellular inhibitor of apoptosis 1), and cdh1 are shown. Amino acid residues involved in post-translational modifications are indicated. E2F1 is subjected to several post-translation modifications in response to genotoxic stress, including phosphorylation on S^31^ by the ataxia telangiectasia mutated/ataxia telangiestasia and Rad3-related (ATM/ATR) [37], on S^364^ by checkpoint kinase 2 (chk2) [38], methylation on K^185^ by Set7/9 [39,40], and on R^109^ by the protein arginine N-methyltransferase 1 (PRMT1) [41], demethylation on R^111/113^ [41], de-neddylation [42], and acetylation by the histone acetyl-transferase p300/CREB-binding protein-associated factor (P/CAF) on K^117/120/125^ [43,44] and K63-ubiquitination on K^161/164^ by cIAP1 [35]. The amino acid residues R^111/113^ are targets of methylation by PRMT5 [41,45], K^117/120/125^ of neddylation [46], K^266^ of sumoylation [47], S^332/337^ of phosphorylation by the cyclin-dependent kinase 1 (cdk1) [48], and S^403^/T^433^ by the p38 mitogen-activated protein kinase (MAPK) [49]. DBD: DNA binding domain.

E2F1 transcription factor is a fundamental regulator of cell cycle progression, allowing the transcription of genes required for the G1 to S phase transition and DNA replication (e.g., *CCNE*, *CCNA*, *DHRF*, *POLA1*, *TK1*) [14]. Accordingly, both protein abundance and activity rapidly rise in quiescent cells in response to mitotic stimulation, as cells re-enter the cell cycle. E2F1 expression levels peak in the late G1 phase to promote S-phase transition, then decrease as cells progress through the S phase and are kept at a low level until the next G1-S (Figure 1). In G0, E2F1 is repressed by binding to a hypophosphorylated form of Rb. Phosphorylation of Rb at the end of the G1 phase releases E2F1, allowing its activation and S phase transition. In late S, the CDK2/Cyclin A complex binds and phosphorylates E2F1, promoting its dissociation from DNA and its downregulation. Importantly, downregulation of E2F1 at the end of the S phase is critical for cells to enter into G2 [15]. Thus, this temporal regulation of E2F1 is essential to preserve the correct timing of cell cycle phase transition. The role of the ubiquitin–proteasome system (UPS) in contributing to the downregulation of E2F1 in the late S phase was suggested as early as 1996 in studies demonstrating that E2F1 can be degraded by the proteasome system [16,17]. Curiously, besides inhibiting E2F1 activity, the binding of Rb protects E2F1 from UPS-mediated degradation [16,17,18,19], suggesting that sequestration of E2F1 by Rb during the G1 phase allows accumulation of a sufficient amount of E2F1 to enable the transcription of genes required for S phase transition. The deletion of the E2F1 carboxyl terminus that overlaps with the Rb binding domain prevents E2F1 ubiquitination [16,18]. Since this region does not contain a ubiquitin acceptor lysine residue, this suggests that it could contain a binding domain for an E3-ubiquitin ligase that could compete with Rb for E2F1 binding [18]. One candidate is the cdh1 co-activator of the anaphase promoting complex/cyclosome (APC/C) E3–ligase complex that was found to inhibit E2F1–Rb binding [20]. Accordingly, mapping of the cdh1-interacting region in the E2F1 protein sequence identified the C-terminal 359–437 amino acid sequence [21] (Figure 2). APC/C is a large multi-subunit E3-ubiquitin ligase that plays a critical role in chromosome segregation during mitosis, mitosis exit and establishment, and maintenance of the G1 phase of cell cycle thanks to its ability to induce ubiquitination of key mediators and regulators of this processes [22,23]. APC/C activation and substrate recognition require the co-activators cdc20 or cdh1. Both have been shown to interact with and promote proteasome-mediated degradation of E2F1 [21,24]. Depletion of cdc20 induces an accumulation of E2F1 during the metaphase [24], whereas inactivation of cdh1 leads to premature S phase entry in Rb-deficient cells or senescence in Rb-expressing fibroblasts [20]. Thus, APC/C^cdc20^ and APC/C^cdh1^ contribute to maintaining low E2F1 protein expression levels during the M and G1 phase of the cell cycle, respectively (Figure 1).

The main candidate able to conjugate ubiquitin chains that target E2F1 for the S-phase specific destruction is the S-phase kinase-associated protein 1 (skp1)–Cullinn1–F-box (SCF)^skp2^ complex. SCF complex is an E3-ligase composed of the scaffold protein Cullin1, the RING protein RBX1, the linker skp1, and an F-box protein that supports the substrate recognition [23]. Skp2 is an F-box, identified because of its capacity to associate with the S-phase kinase CDK2/cyclin A [25]. The downregulation of skp2 prevents S phase transition, demonstrating the critical role of this protein in G1–S phase transition [25]. Skp2 expression fluctuates in a cell-cycle-dependent manner: it is detected in G1/S, accumulates along the G2/M, and then drastically falls as the cells exit mitosis. Skp2 binds the amino acid residues 1–76 of E2F1 (Figure 2) and promotes E2F1 degradation by the proteasome [26]. Expression of an E2F1-deletion mutant devoid of the skp2-binding region leads to a prolonged S phase, demonstrating the importance of skp2 in mediating downregulation of E2F1 prior to S phase exit [26]. Of interest, (i) *skp2* gene expression is positively regulated by E2F1 during the S-phase [27], and (ii) skp2 protein is regulated by Rb, which favors its APC/C^cdh1^-mediated degradation in G1 [28], thus participating in the overall accumulation of E2F1. In addition to Rb, several cell cycle regulators have been shown to interfere with the UPS-mediated degradation of E2F1, including the tumor suppressor p19^ARF^, which binds E2F1 and favors its degradation [29,30], or the murine double minute 2 (MDM2), which directly binds E2F1 and prevents skp2-mediated E2F1 degradation [31]. Since ubiquitination is a reversible event, E2F1 stability is also logically controlled by DUBs. Among the three E2F1 DUBS currently known, two can affect E2F1 stability: POH1 (also known as PSMD14: 26s proteasome non-ATPase regulatory subunit 14) [32] and Cezanne, whose depletion favors E2F1 downregulation and compromises the S-phase transition [33].

The nature of ubiquitin chains as well as the E2F1 lysine acceptors of ubiquitins has not been much studied. Modifications of E2F1 with K11-, K48-, and K63-linked ubiquitin chains have been reported [21,32,34,35]. The APC/C E3-ligase complex generally promotes the conjugation of K11-linked ubiquitin chains by using the E2 ubiquitin conjugating enzyme 2C (Ube2C) and 2S (Ube2S). Accordingly, the APC/C^cdh1^ was found to conjugate K11-linked ubiquitin chains on E2F1 [21]. However, a recent report demonstrated that APC/C can conjugate K11/K48-branched ubiquitin chains that produce a strong proteolytic signal [36]. Whether E2F1 can be modified by such ubiquitin chains has not been investigated. Whereas the K11- and K48-linked ubiquitin chains are most often known as degradation signals, K63-linked ubiquitin chains are generally associated with non-degradative processes. Several pieces of evidence suggest that E2F1 could also be regulated by non-degradative ubiquitination events. Unlike Cezanne or POH1, the DUB Uch37 (Ubiquitin carboxy-terminal hydrolase 37) that removes K63-linked ubiquitin chains does not affect E2F1 stability but its transcriptional activity [34]. A non-degradative K63-poly-ubiquitination of E2F1 has been recently reported in the S phase of the cell cycle, associated with a stabilization of the protein and a maximal activity (Figure 1) [35]. The K63-linked ubiquitin chain conjugation occurs on lysine cluster 161/164, located in the DNA binding domain. Mutation of these lysine residues abrogates the capacity of E2F1 to modulate cell proliferation. The inhibitor of apoptosis (IAP) family member cIAP1 has been proposed as the E3-ligase able to promote such ubiquitination [35]. Moreover, cIAP1 was found to compete with Rb for E2F1 binding and its overexpression enhances Rb phosphorylation, suggesting that cIAP1-mediated K63 ubiquitination of E2F1 could be a signal for the activation of E2F1 in the early S phase of the cell cycle.

## 4. Role of Ubiquitination in the Regulation of E2F1 upon DNA Damage

Accumulation of E2F1 is an early event during the DNA damage response, contributing to cell cycle arrest and apoptosis. The molecular mechanisms involved in the upregulation of E2F1 have been extensively studied and involve its phosphorylation on S31 by the ataxia telangiectasia mutated/ataxia telangiectasia and Rad3-related (ATM/ATR) kinases [37] and on S364 by the checkpoint kinase 2 (chk2) [11,38] (Figure 2). Phosphorylation at S31 is a signal for binding of the chaperon 14-3-3τ, which promotes stabilization of E2F1 through the inhibition of degradative ubiquitination [50]. Other post-translational modifications of E2F1 that contribute to E2F1 stabilization and activation in response to genotoxic stress include acetylation [43,44,51], demethylation [39,40], arginine methylation [41], and de-neddylation [42] (Figure 2). In 2005, Galbiati et al. observed that accumulation of E2F1 in response to the topo-isomerase 1 inhibitor camptothecin was associated with ubiquitination, independently of SCF^skp2^ [44]. More recently, the DNA damaging agent etoposide was reported to induce K63-linked poly-ubiquitin chains on E2F1 K161/K164 in a cIAP1-dependent manner [35]. cIAP1-mediated E2F1 ubiquitination required an arginine methylation. Mutation of lysine residues 161 and 164 into arginine abolished the DNA-damage-induced stabilization of E2F1. Mutation of these lysine residues as well as the downregulation of cIAP1 also completely prevented the recruitment of E2F1 on the promoters of E2F target genes [35,52], suggesting that these K63-linkage-specific chains could provide a signal for chromatin anchorage. This hypothesis is strengthened by the observation of Mahanic et al*.* of K63-ubiquitinated E2F1 on the chromatin [34]. Moreover, the de-ubiquitinating enzyme UCH37, which removes K63-specific ubiquitin linkages from E2F1, was found to interact with E2F1 upon DNA stimulation at the site of transcriptional regulation. However, depletion of UCH37 decreases E2F1’s transcriptional activity, suggesting the presence of K63-linked ubiquitin chains that are detrimental to E2F1 activity. However, in this study, the lysine residues involved have not been determined. Since the protein sequence of E2F1 harbors 14 lysine residues, it is likely that K63-linkage specific ubiquitin chains may bind different lysine residues, providing signals that activate different intracellular signaling pathways.

## 5. Conclusions

While the regulation of E2F1 by ubiquitination was suggested early on, the E3-ubiquitin ligase involved, the nature of ubiquitin chains, and the acceptor Lysine residues are still not well determined in most cases. E2F1 harbors 14 lysine residues that are all potential acceptors for ubiquitin modification. E2F1’s abundance fluctuates along the cell cycle and in response to DNA damage, but also in response to differentiating agents. For example, during keratinocyte differentiation, E2F1 is exported from the nucleus to the cytoplasm, where it undergoes degradation in a UPS-dependent manner [53]. Moreover, non-transcriptional activities of E2F1 have been described that could be associated with nuclear export. Future research will likely determine the role of ubiquitination in these processes.

The complexity and variability of ubiquitin signals make ubiquitination a critical mechanism of regulation of the cellular fate of intracellular proteins. Ubiquitination and deubiquitination can be implemented very quickly to allow fast cellular adaption to multiple variations. The Rb-E2F1 axis is an important cellular pathway that regulates normal cell proliferation. Increasing evidence shows that this pathway is dysregulated in a large proportion of human malignancies. Since both Rb and E2F1 are regulated by ubiquitination, dysregulation of this post-translational modification could serve as a marker for cancer development. Alternatively, a better understanding of the molecular players may help in treating patients suffering from cancer as unexpected targets may be unveiled, allowing restoration of a proper Rb-E2F1 axis in cancer cells.

## Abbreviations

APC/C	Anaphase promoting complex/cyclosome
ATM	Ataxia telangiectasia mutated
ATR	Ataxia telangiectasia and Rad3-related
*CCNE*	Cyclin E gene
*CCNA*	Cyclin A gene
CDK2	Cyclin-dependent kinase 2
chk2	Checkpoint kinase 2
cIAP1	Cellular inhibitor of apoptosis 1
*DHRF*	Dihydrofolate reductase
DBD	DNA binding domain
DP1	Dimerization partner 1
DUBs	Deubiquitylases
E2F	E2 promoter binding factor
MAPK	mitogen-activated protein kinase
MDM2	Murine double minute 2
PRMT	protein arginine *N*-methyltransferase
PSMD14	26S proteasome non-ATPase regulatory subunit 14
Rb	Retinoblastoma
Skp	S-phase kinase associated protein
SCF	Skp1 (S-phase kinase associated protein 1)-Cullinn1-F-box
Ube	ubiquitin conjugating enzyme
UBDs	Ubiquitin-binding domains
Uch37	Ubiquitin C-terminal hydrolase 37
UPS	Ubiquitin-proteasome system
